# Matters of the heart; antibiotic prophylaxis for prevention of infective endocarditis—are we getting it right?

**DOI:** 10.1038/s41432-025-01185-w

**Published:** 2025-08-28

**Authors:** Rosie Fletcher, Adam Jones

**Affiliations:** 1 Specialty Registrar in Oral Surgery, Leeds Dental Institute, Leeds, UK; 2 Clinical Lecturer and Specialist in Oral Surgery, Leeds Dental Institute, Leeds, UK

**Keywords:** Dentistry, Oral microbiology, Oral medicine

## Abstract

**A Commentary on:**

**Sperotto F, France K, Gobbo M** **et al**.

Antibiotic prophylaxis and infective endocarditis incidence following invasive dental procedures: a systematic review and meta-analysis. *JAMA Cardiol* 2024; 9:599. 10.1001/jamacardio.2024.0873.

**Objectives:**

This systematic review evaluates the association between antibiotic prophylaxis (AP) and the incidence of infective endocarditis (IE) following invasive dental procedures (IDPs).

**Materials and methods:**

A systematic search was conducted across PubMed, Cochrane-CENTRAL, Scopus, Web of Science, Proquest, and Embase, from inception to May 2023. Observational studies, including case-control, case-crossover, cohort, self-controlled case-series, and time-trend studies were included. Data were extracted independently, and structured tools were used to evaluate study quality. A random-effects meta-analysis estimated the pooled-relative risk (RR) of developing IE in high-risk subjects who received AP compared to those who did not.

**Results:**

Of 11,217 identified records, 30 studies met inclusion criteria, comprising 1,152,345 IE cases. Among 12 relevant studies, five found a significant protective effect of AP in high-risk subjects. Four studies were combined in meta-analysis and showed AP was associated with a significantly lower IE risk in high-risk individuals (pooled-RR = 0.41, 95% CI: 0.29–0.57). No significant association was found for moderate- or low/unknown-risk subjects. Time-trend studies showed mixed results: some indicated increased IE incidence after AP guideline changes, while others found no change or a decrease.

**Conclusions:**

Despite limitations, this review provides an important update on AP use in preventing IE after IDPs. Evidence supports AP use for high-risk individuals, while data remain inconclusive for moderate-risk populations, highlighting the need for further research.

## GRADE Rating:



## Commentary

The role of AP in preventing IE following IDPs remains debated [1, 2, 3]. This systematic review and meta-analysis by Sperotto et al. [4] explore whether AP reduces IE risk in different patient groups.

This study is particularly relevant in the UK, where NICE recommends against routine AP for dental procedures [5], contrasting with American Heart Association (AHA) and European Society of Cardiology (ESC) guidance supporting AP for high-risk patients [6, 7]. With IE incidence rising, UK dentists must balance patient safety and antimicrobial stewardship. This commentary explores implications for dental professionals.

### Context and controversy

IE is a rare but serious condition, often caused by viridians group streptococci entering the bloodstream [8, 9, 10]. Dental procedures disrupting oral tissues can cause transient bacteraemia, potentially contributing to IE in susceptible individuals.

Before 2008, UK guidance aligned with AHA and ESC recommendations, routinely prescribing AP to at-risk patients [6, 7]. NICE changed this in 2008, recommending against routine AP due to insufficient evidence of benefit, antimicrobial resistance concerns, and potential risks such as anaphylaxis [5]. Some studies suggest increased IE incidence following this change [11–13]; others do not [14–16].

This study [4] adds to the debate, with findings supporting AP use in high-risk patients, but not in moderate- or low-risk groups. However, inconsistent time-trend data and lack of randomised trials mean uncertainty remains.

### Strengths and limitations of the study

This study’s strengths include a large dataset and structured quality assessments. Meta-analysis of case-crossover and cohort studies strengthens the argument that AP reduces IE risk in high-risk patients.

However, observational studies are prone to confounding, and RCTs are unlikely in this field and so establishment of a direct causal link remains challenging. Time-trend findings were inconsistent, possibly reflecting differences in healthcare systems, populations, guideline adherence, or IE causative organisms [16, 17]. Other factors like improved diagnostics, increased prosthetic heart valve use, and an ageing population may also influence IE trends independently of AP practices.

### Clinical implications for UK dentists

In October 2024, NICE issued “Exceptional surveillance of prophylaxis against infective endocarditis,” [18] which reviewed existing evidence, including Sperotto et al. [4]. It concluded that robust research is lacking but limited findings indicate invasive dental treatments might contribute to a small proportion of IE in high-risk individuals. NICE CG64 guidance remained unchanged but now signposts SDCEP implementation advice to help clarify those patients that require special consideration for antibiotic prophylaxis [19]. SDCEP is currently reviewing its own guidance.

Considering current evidence dentists should:

1. Identify patients at increased risk and differentiate those requiring special consideration
  While NICE guidance does not recommend routine AP, it allows for individual clinical judgment. SDCEP [19] advises increased-risk patients include those with:
  Heart disease that has developed over time, involving stenosis or regurgitation
  Hypertrophic cardiomyopathy
  History of IE
  Certain structural heart defects (excluding isolated or fully repaired cases)
  Valve replacements
  Within this group, a subset requires special consideration, including:
  Prosthetic heart valves, including transcatheter valves, or prosthetic material used in valve repair
  History of Previous IE
  Certain types of congenital heart disease
  Cardiac transplant recipients with valvulopathy
  Care for these patients should involve their cardiologist or relevant medical specialist if IDPs are planned.
2. Understand which procedures are invasive
  AP is considered for procedures likely to cause significant bacteraemia, including [19]:
  Extractions
  Periodontal surgery or subgingival scaling
  Implant placement
  Endodontic treatment before apical stop establishment
  Any mucosal incision
  Procedures like supragingival restorations, BPE, scaling above the gumline, orthodontics, and radiographs are non-invasive and do not require AP [19].
3. Communicate effectively with medical colleagues
  UK guidance differs from other countries, so clear communication is essential. If a cardiologist recommends AP, dentists should document and respect this if appropriate. Patients should be fully informed of the risks and benefits when AP is considered [19].
4. Promote antimicrobial stewardship and educate patients

Dentists must minimise unnecessary antibiotic use to combat resistance and adverse effects. When AP isn’t indicated, patients should be encouraged to maintain good oral hygiene and understand IE signs and symptoms.

## Conclusion

For UK dentists, the study highlights the importance of adhering to NICE guidelines alongside SDCEP implementation advice while considering individual patient circumstances. Clinicians should be confident in avoiding unnecessary AP prescriptions but remain open to case-by-case decision-making in collaboration with patients and medical colleagues.

While the debate over AP in dentistry continues, this study is a valuable addition to the evidence base for clinical decision-making.

Practice points

- Identify high-risk patients: Those requiring special consideration include individuals with prosthetic valves, previous IE, specific congenital heart defects, or cardiac transplant-related valvulopathy.
- Know which procedures require AP: Only invasive dental procedures that manipulate gingival or mucosal tissues should prompt consideration of AP.
- Prioritise antimicrobial stewardship and education: Antimicrobial stewardship remains important, and patient education on maintaining good dental health, oral hygiene, and recognising IE signs and symptoms should be prioritised as a key IE reduction strategy.
